# Efficient Near-Infrared-Activated Photocatalytic Hydrogen Evolution from Ammonia Borane with Core-Shell Upconversion-Semiconductor Hybrid Nanostructures

**DOI:** 10.3390/nano11123237

**Published:** 2021-11-29

**Authors:** Andrew J. Evangelista, Mariia Ivanchenko, Hao Jing

**Affiliations:** Department of Chemistry and Biochemistry, George Mason University, Fairfax, VA 22030, USA; aevange3@gmu.edu (A.J.E.); mivanch@gmu.edu (M.I.)

**Keywords:** near-infrared, hydrogen evolution, upconversion, semiconductor, core-shell

## Abstract

In this work, the photocatalytic hydrogen evolution from ammonia borane under near-infrared laser irradiation at ambient temperature was demonstrated by using the novel core-shell upconversion-semiconductor hybrid nanostructures (NaGdF_4_:Yb^3+^/Er^3+^@NaGdF_4_@Cu_2_O). The particles were successfully synthesized in a final concentration of 10 mg/mL. The particles were characterized via high resolution transmission electron microscopy (HRTEM), photoluminescence, energy dispersive X-ray analysis (EDAX), and powder X-ray diffraction. The near-infrared-driven photocatalytic activities of such hybrid nanoparticles are remarkably higher than that with bare upconversion nanoparticles (UCNPs) under the same irradiation. The upconverted photoluminescence of UCNPs efficiently reabsorbed by Cu_2_O promotes the charge separation in the semiconducting shell, and facilitates the formation of photoinduced electrons and hydroxyl radicals generated via the reaction between H_2_O and holes. Both serve as reactive species on the dissociation of the weak B-N bond in an aqueous medium, to produce hydrogen under near-infrared excitation, resulting in enhanced photocatalytic activities. The photocatalyst of NaGdF_4_:Yb^3+^/Er^3+^@NaGdF_4_@Cu_2_O (UCNPs@Cu_2_O) suffered no loss of efficacy after several cycles. This work sheds light on the rational design of near-infrared-activated photocatalysts, and can be used as a proof-of-concept for on-board hydrogen generation from ammonia borane under near-infrared illumination, with the aim of green energy suppliers.

## 1. Introduction

Due to the extensive production of air-based pollution and the ultimately limited supply of fossil fuels, a search for a suitable fuel replacement is a priority. Switching to a clean source of energy is urgent because of the amount of damage the atmosphere has already suffered. In the search to replace fossil fuels with a cleaner alternative, hydrogen has emerged as a high potential candidate. Hydrogen is attractive due to its high chemical energy density and its ability to be stored in the three phases of matter: solid, liquid, and gas. Additionally, when produced from a clean and renewable source, the hydrogen producing reaction can have virtually zero emissions [1].

Owing to the explosive nature of gaseous hydrogen, and the immense pressure and sealing necessary to store liquid hydrogen, solid storage materials have gained significant traction [2,3,4]. In particular, chemically stored hydrogen, such as solid hydride complexes, specifically borohydride complexes, have been theorized as a possible solution [5,6,7]. For a while, sodium borohydride was the most popular choice for solid storage, since it could be used as an aqueous fuel independently, in a direct fuel cell, or direct liquid-free cell [8,9]. However, in 2007, the United States Department of Energy recommended that sodium borohydride not be used for on-board applications, such as for an automobile or a portable power pack [10]. Instead, ammonia borane (AB) was identified as a promising alternative, by virtue of its remarkable gravimetric hydrogen storage capacity of 19.5 wt % and high stability in solid state at ambient temperature [11,12]. The catalytic decomposition of ammonia borane to produce hydrogen has drawn much attention in recent years [13,14,15,16,17,18,19,20,21,22,23,24,25]. Although various approaches were used to release hydrogen from AB over the years [26,27,28,29,30,31,32,33,34,35,36,37], there has been little that allows the claim of a practical application, due to the harsh conditions, such as high temperature during thermolysis and slow kinetics of dehydrogenation. To circumvent these issues, the exploitation of environmentally friendly technologies becomes imperative in the modern climate. Compared to other approaches, photocatalysis is superior, and is widely applied in photodegradation and photo-oxidization, as it utilizes the inexhaustible solar energy, while not generating hazardous pollutants. Inspired by the Fujishima Honda Effect reported in 1972 [38], a plethora of semiconductor-based photocatalysts, such as TiO_2_, g-C_3_N_4_, CdS [39], various perovskites [40,41,42,43,44,45,46,47,48,49,50,51,52,53,54,55], and WO_3_/rGO [56] have been utilized in hydrogen production. Additionally, non-semiconductor catalysts based on metals including platinium [57], rhenium [58], ruthenium [59,60], and iridium [61] have also been used in the catalytic generation of H_2_ [62,63]. For these photocatalysts with their light absorption threshold confined in either ultraviolet (UV, 300–400 nm) or visible (VIS, 400–700 nm) region [64], the major limitation is that only UV and/or visible light photons can be utilized, which accounts for 5% and 43% of the full solar spectrum, respectively. The under-exploitation of more abundant low-energy near-infrared (NIR) light, which makes up 52% of the solar energy, undoubtedly imposes intrinsic limitations to the maximum achievable solar energy conversion efficiency, and hinders the practical application in the field of solar-to-fuels energy conversion. Although metallic or multi-metallic nanoparticles with geometrically tunable localized surface plasmon resonances (LSPRs) redshifted to NIR region, materials including ruthenium, platinum, palladium, cobalt, iron, nickel, silver [65,66,67,68,69,70,71,72,73,74,75], cadmium and tungsten [76], gold [77], and copper [78,79,80,81,82] were used for efficient H_2_ generation; the inherent photo-corrosion susceptibility and inevitable high processing cost make their performance less satisfactory. Therefore, it is of great significance to design and develop more efficient photocatalysts with capabilities to utilize NIR light energy for stable photocatalytic H_2_ production.

One promising tactic to improve the utilization of the low-energy NIR light to drive H_2_ evolution is coupling nonlinear optical materials that can strongly absorb NIR light photons with a photocatalytic semiconductor. Among them, lanthanide-doped upconversion nanoparticles with well-controlled structures and morphologies are considered efficient candidates due to their unique and remarkable optical characteristics, such as their ability to convert near-infrared incident light into high-energy ultraviolet or visible light photons, large anti-Stokes shifts, sharp and tunable multi-peak line emission profiles, and excellent photostability [83,84,85,86,87,88]. Subsequently, the upconverted light photons need to be reabsorbed by the appropriate semiconductors, with a concomitant injection of electrons from the valance band (VB) to the conduction band (CB) of the semiconductor to generate photoinduced carriers. In this work, for the first time, we report the synthesis and characterization of core-shell UCNPs-semiconductor (NaGdF_4_: Yb^3+^/Er^3+^@ NaGdF_4_ @Cu_2_O) hybrid nanostructures, and demonstrate the NIR-driven photocatalytic activities for H_2_ evolution from ammonia borane (AB) molecules. Cuprous oxide (Cu_2_O), a p-type semiconductor with high optical absorption coefficients, has a bulk band gap of ~2.2 eV [89,90,91]. This interesting exitonic feature ensures the spectral overlapping between absorption of Cu_2_O and emission bands of UCNPs, which results in efficient energy transfer from UCNPs to Cu_2_O through the reabsorbing of upconverted light photons. Integrating UCNPs and Cu_2_O into one nano-entity with core-shell morphology efficiently promotes the direct energy migration under NIR light irradiation for the formation of photoinduced charge carriers, due to the intimate contact of two distinct materials. We can envision that this paradigm will provide new insights for the rational design of NIR-responsive photocatalysts, and reveal a new way for exploitation of sustainable energy sources.

## 2. Materials and Methods

### 2.1. Materials

Gadolinium (III) acetate hydrate (99.9%) (Gd(OAc)_3_), ytterbium (III) acetate hydrate (99.9%) (Yb(OAc)_3_), erbium (III) acetate hydrate (99.9%) (Er(OAc)_3_), oleic acid (90%) (OA), 1-octadecene (90%) (1-ODE), sodium hydroxide (≥98%) (NaOH), ammonium fluoride (99.9%) (NH_4_F), methanol (99.9%) (MeOH), cyclohexane (99.5%) (CH), nitrosyl tetrafluoroborate (95%) (NOBF_4_), chloroform (99%) (CHCl_3_), dimethylformamide (99.8%) (DMF), copper (II) nitrate (99.999%) (Cu(NO_3_)_2_), hydrazine (35 wt % in H_2_O) (N_2_H_4_), 2-propanol (C_3_H_8_O), and ammonia borane (90%) (AB) were all purchased from Sigma Aldrich (St. Louis, MO, USA) and used without further purification. Ethanol (EtOH) was purchased from VWR and used as is. Ultrapure water (18.2 MΩ·cm resistivity, PURELAB Ultra Ionic Polishing system) was used for all experiments.

### 2.2. Characterization

The size and morphology of the nanoparticles were determined by using a Titan 80–300 analytical transmission electron microscope (TEM) (FEI Company, Hillsboro, OR, USA) operating at 300 kV. The standard TEM samples were prepared by dropping solutions of nanoparticles onto the surface of copper grids. Upconversion luminescence (UCL) spectra were recorded at room temperature on a high sensitivity QE Pro-FL spectrofluorometer (Ocean Optics, Inc, Dunedin, FL, USA), with the excitation source of an external 0–5 W adjustable continuous wave 980 nm laser diode (Dragon Lasers, China). Hydrogen evolution was tracked using a HP 5890 gas chromatograph (Hewlett-Packard, Palo Alto, CA, USA), equipped with a molecular sieve column and a thermal conductivity detector (TCD). The column had a 15 m length, a 0.530 mm diameter, and a 25.0 μm film. Energy dispersive X-ray spectroscopy (EDX) measurements were obtained on JSM-IT500HR scanning electron microscope (SEM) (JEOL USA, Inc, Peabody, MA, USA) equipped with an EDAX APEX detector (AMETEK, Inc., Berwyn, PA, USA). Powder X-ray diffraction (XRD) of the sample was measured at room temperature for 2 h with the scattering angle 2θ range from 20° to 80°, using a benchtop Miniflex-600 powder X-ray diffractometer (Cu Kα, λ = 1.5418 Å) (Rigaku Americas Corporation, The Woodlands, TX, USA). Size distribution/histogram was obtained using ImageJ software after measuring ~100 nanoparticles.

### 2.3. Photocatalytic H_2_ Evolution

First, the semiconductor-coated UCNPs (0.5 mL, 1.26 mL, 2 mL) were added to a solution of ammonia borane (10 mL, 0.1 M) in a glass photoreactor equipped with a water jacket and quartz top. The water circulating in the jacket was kept at 20 °C to prevent the thermal decomposition of the ammonia borane. The solution was degassed with argon for 30 min. The photoreactor was then sealed and a 980 nm laser was introduced at 1 W. A 100 μL sample was taken every 30 min, for 180 min. To study the roles of hydroxyl radicals in the photcatalytic evolution of H_2_, 2-propanol (0.1 mL) was added to the mixture in the presence of 2.0 mL semiconductor-coated UCNPs and 10 mL 0.1 M ammonium borane. Other conditions were the same as mentioned above for the photocatalysis.

### 2.4. Synthesis of NaGdF_4_:Yb^3+^/Er^3+^ UCNPs

First, Gd(OAc)_3_ (67 mg), Yb(OAc)_3_ (83 mg), Er(OAc)_3_ (4.6 mg), oleic acid (4 mL) and 1-ODE (6 mL) were added to a 50 mL 3-neck flask. The flask was put under argon protection and heated to 150 °C with magnetic stirring. At temperature, vacuum was pulled to remove oxygen, moisture, and other low boiling point impurities. After several degas cycles, the reaction was cooled to 50 °C. At this point, a mixture of NaOH (50 mg) and NH_4_F (70 mg) dissolved in 10 mL of methanol was added and stirred for 30 min. Next, the solution was degassed for several cycles at various temperatures (70 °C, 100 °C, 150 °C) to remove the methanol, and any other lower boiling point impurities. The reaction solution was then heated to 290 °C, and allowed to react for 90 min. After cooling, the mixture was spun in a centrifuge at 6000 rpm for 4 min and washed once with ethanol (5 mL), and then spun again and washed with a mixture of ethanol (8 mL) and cyclohexane (4 mL). After a final spin, the particles were redispersed in cyclohexane (4 mL).

### 2.5. Synthesis of Hydrophobic Core-Shell NaGdF_4_:Yb^3+^/Er^3+^@NaGdF_4_ UCNPs

To start, the previously synthesized cores NaGdF_4_:Yb^3+^/Er^3+^, in cyclohexane (4 mL), were added to a 50 mL 3-neck flask, along with Gd(OAc)_3_ (134 mg), oleic acid (4 mL), and 1-ODE (6 mL). The flask was put under argon protection with magnetic stirring, and the mixture was degassed at 150 °C. Once the reaction naturally cooled to 50 °C, both NaOH (1 mL, 1.0 M in methanol) and NH_4_F (3.33 mL, 0.4 M in methanol) were added. The reaction mixture was stirred for 30 min and was then degassed again at 70 °C, 100 °C, and 150 °C, to remove methanol and other low boiling point impurities. The temperature was increased to 290 °C, and the reaction was allowed to stir for 90 min. Upon cooling to room temperature, the reaction solution was washed by the same procedure as the core particles. The final dispersion was in 4 mL of cyclohexane.

### 2.6. Transformation to Hydrophilic NaGdF_4_:Yb^3+^/Er^3+^@NaGdF_4_ UCNPs

The core/shell UCNPs (NaGdF_4_:Yb^3+^/Er^3+^@NaGdF_4_), in cyclohexane (2 mL), were mixed with NOBF_4_ (2 mL, 50 mM in DMF). The combination was sonicated for 30 min, and the layers were allowed to separate. The cyclohexane layer was removed, and chloroform (8 mL) was added to the hydrophilic layer. The solution was centrifuged at 6000 rpm for 5 min. The pellet was then redispersed in 2 mL of DMF.

### 2.7. Synthesis of NaGdF_4_:Yb^3+^/Er^3+^@NaGdF_4_@Cu_2_O UCNPs

The hydrophilic UCNPs (NaGdF_4_:Yb^3+^/Er^3+^@NaGdF_4_), in DMF (2 mL), were added to a reaction vial with magnetic stirring. A 25 mM solution of Cu(NO_3_)_2_ (3.2 mL) was added dropwise, and the mixture stirred for 30 min. Next, NaOH (4.8 mL, 0.1 M) was added quickly, and the reaction was stirred for 30 more minutes. Then, N_2_H_4_ (4.8 mL, 0.1 M) was added dropwise, and the solution was allowed to stir for an additional 30 min. The reaction solution was transferred to an autoclave reactor with a Teflon liner, and was heated to 170 °C for 18 h. The solution was then spun in a centrifuge at 3000 rpm for 1 min to remove large particles, and then the supernatant was allowed to settle overnight. The remaining solution was then spun at 12,000 rpm for 20 min, and the pellet was redispersed in ultra-pure water (1 mL, 18 MΩ cm).

## 3. Results

**Figure 1:**
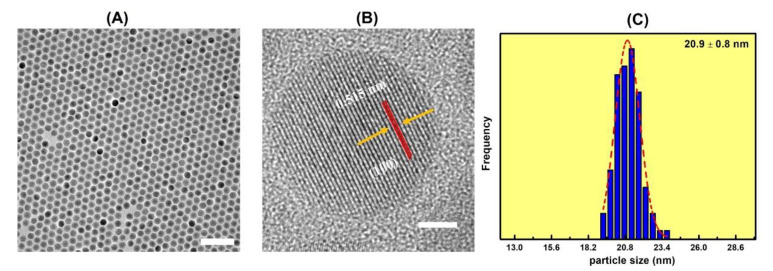
(**A**) A TEM image of NaGdF_4_:Yb^3+^/Er^3+^ particles, where both the hexagonal shape of the formation and the particles is evident. The scale bar is 100 nm. (**B**) An HRTEM image of a select NaGdF_4_:Yb^3+^/Er^3+^ particle, where the lattice fringes can be seen. The d-spacing was measured to be 0.515 nm, corresponding to the (100) face of β-NaGdF_4_:Yb^3+^/Er^3+^. The scale bar is 5 nm. (**C**) The size distribution diagram of the NaGdF_4_:Yb^3+^/Er^3+^ particles, measured from ~100 particles.

**Figure 2:**
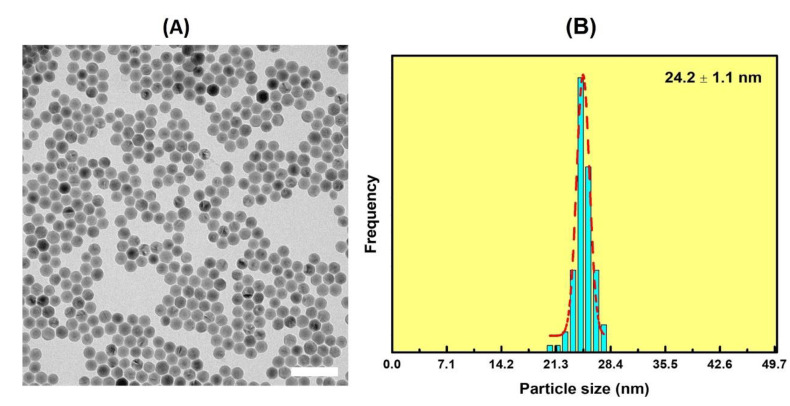
(**A**) A TEM image of NaGdF_4_:Yb^3+^/Er^3+^@NaGdF_4_. The scale bar is 100 nm. (**B**) The size distribution diagram of NaGdF_4_:Yb^3+^/Er^3+^@NaGdF_4_. The increase in size and uniformity can be noted here, due to the addition of the inert NaGdF_4_ shell.

After purification, the UCNPs were measured via TEM at each stage in the synthesis, to ensure uniformity in size and morphology. As shown in Figure 1A, the as-synthesized core UCNPs (NaGdF_4_:Yb^3+^/Er^3+^) were single crystalline and highly monodisperse with spherical morphologies. The lattice fringes had d-spacings of 0.515 nm, corresponding to the (100) planes of ß-phase NaGdF_4_:Yb^3+^/Er^3+^, as demonstrated in Figure 2B. The particle sizes were determined to be 20.9 nm ± 0.8 nm, based on the size distribution diagram obtained by measuring ~100 particles (Figure 1C). After epitaxially growing an inert shell of NaGdF_4_ on the spherical core UCNPs, the sizes of core-shell UCNPs (NaGdF_4_:Yb^3+^/Er^3+^@ NaGdF_4_) increased slightly to 24.2 nm ± 1.1 nm, and the shapes and morphologies evolved to hexagons, confirmed by TEM images shown in Figure 2.

**Figure 3:**
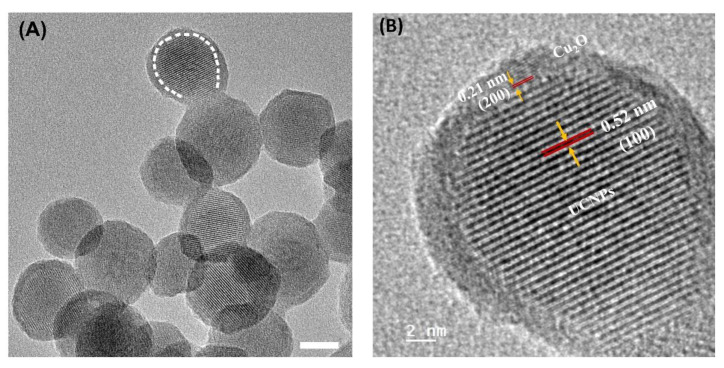
High resolution TEM images of (**A**) NaGdF_4_: Yb^3+/^Er^3+^@ NaGdF_4_ @Cu_2_O, with the boundary between UCNPs and Cu_2_O clearly observed and highlighted by a circular white dashed line and (**B**) a zoom-in of the particle outlined in (**A**). The fringes can be seen and a visual differentiation between the NaGdF_4_ and the Cu_2_O can be noted. Additionally, the d-spacing of each can be measured. They are 0.52 and 0.21, which correspond to the (100) facet of NaGdF_4_ and (200) crystal plane of Cu_2_O, respectively. The scale bars are (**A**) 10 nm and (**B**) 2 nm, respectively.

Subsequently, the thin layer of Cu_2_O was successfully grown on the surface of ß-NaGdF_4_:Yb^3+^/Er^3+^@NaGdF_4_ to form eccentric core-shell UCNP-semiconductor hybrid hetero-nanostructures (NaGdF_4_:Yb^3+^/Er^3+^@NaGdF_4_@Cu_2_O), with a facile and robust wet-chemistry approach, using an autoclave reactor with a Teflon liner, at a temperature of 170 °C for 18 h. The detailed structural information on ß-NaGdF_4_:Yb^3+^/Er^3+^@NaGdF_4_, the Cu_2_O shell, as well as the interfaces between the core and shell, were clearly provided by the HRTEM measurements. As shown in Figure 3A, the shapes of the hybrid nanostructures changed from hexagons to quasi-spheres after Cu_2_O was grown on the surface, and the surface roughness of the core-shell hybrid nanoparticles was clearly observed in the HRTEM image, due to the growth of the Cu_2_O layer. The boundaries between the UCNP cores and the outer layer of Cu_2_O were also resolved and highlighted by the white dashed line. The single zoomed in particle further confirmed the core-shell morphology and the atomically well-defined crystalline facets, with the lattice fringes showing interplanar distances of 0.52 nm and 0.21 nm, corresponding to the (200) planes of UCNPs and Cu_2_O, respectively (Figure 3B). An additional high-resolution TEM image with interplanar spacings of the core and shell was shown (Figure S1) to further confirm the crystallographic planes of NaGdF_4_ and Cu_2_O in the hybrid heteronanostructures. The crystal structures of the hybrid nanoparticles (NaGdF_4_:Yb^3+^/Er^3+^@NaGdF_4_@Cu_2_O) were examined by X-ray powder diffraction (XRD) analysis (Figure S2). The characteristic diffraction peaks indexed to the pure hexagonal phase NaGdF_4_ (JCPDS 27-0699) and Cu_2_O (JCPDS 05-0667) further confirmed the core-shell structure synthesized. Meanwhile, the energy dispersive X-ray (EDX) spectroscopy analysis was performed to validify the chemical compositions (Figure S3).

**Figure 4:**
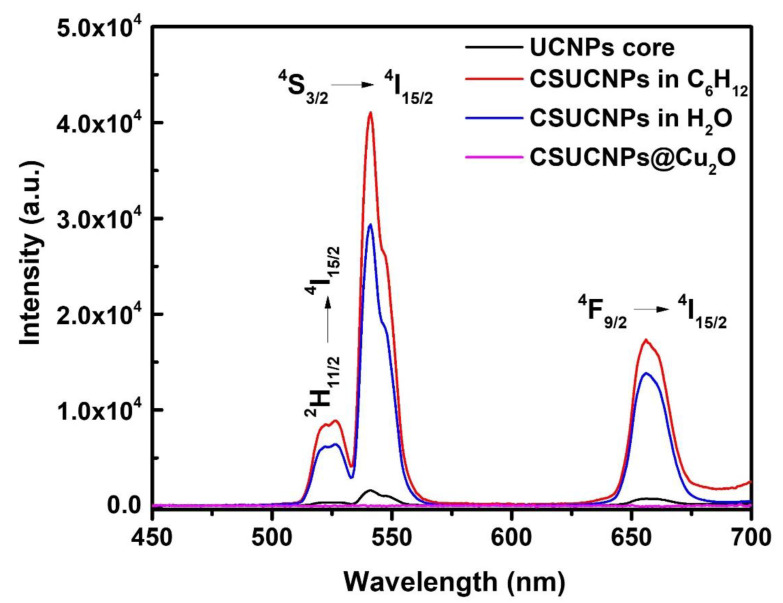
The photoluminescence spectra of the particles at various stages in the synthesis.

Figure 4 shows the upconversion photoluminescence spectra of NaGdF_4_:Yb^3+^/Er^3+^ (UCNPs core), hydrophobic (CSUCNPs in C_6_H_12_) and hydrophilic NaGdF_4_:Yb^3+^/Er^3+^@NaGdF_4_ (CSUCNPs in H_2_O), and NaGdF_4_:Yb^3+^/Er^3+^@NaGdF_4_@Cu_2_O (CSUCNPs@Cu_2_O) hybrid nanostructures, under 980 nm NIR laser excitation. Three distinct non-radiative relaxations in the spectra were observed, including ^2^H_11/2_ → ^4^I_15/2_ (528 nm), ^4^S_3/2_ → ^4^I_15/2_ (541 nm) and ^4^F_9/2_ → ^4^I_15/2_ (656 nm), which was attributed to the transitions of erbium (Er^3+^) ions after the pump photons were absorbed by ytterbium (Yb^3+^) ions, and the energy was resonantly transferred to adjacent Er^3+^ in the matrix under the excitation. The peak intensities of the as-synthesized hydrophobic NaGdF_4_:Yb^3+^/Er^3+^@NaGdF_4_ were significantly increased after an inert layer of NaGdF_4_ was grown, as shown by the red curve in Figure 4. During the transformation from hydrophobic to hydrophilic NaGdF_4_:Yb^3+^/Er^3+^@NaGdF_4_ dispersed in aqueous phase through a facile ligand-exchange method using NOBF_4_, the photoluminescence emission intensities decreased, mainly due to the detrimental quenching effects of the H_2_O molecules demonstrated by the blue curve in Figure 4. Upon the coating of the semiconductor Cu_2_O layer, the photoluminescence of the hybrid NaGdF_4_:Yb^3+^/Er^3+^@NaGdF_4_@Cu_2_O nanoparticles was almost quenched (pink curve), indicating the efficient energy transfer from UCNPs to the shell Cu_2_O under 980 nm excitation. Cu_2_O has a bulk band gap of ~2.2 eV, and its absorption spectrally overlaps with all three emission bands of the UCNPs, resulting in the reabsorption of the upconverted light photons, and the quenching of the upconverted photoluminescence of the core-shell hybrid hetero-nanostructures. It is noteworthy to point out that the intimate contact stemming from the core-shell structures facilitated the reabsorption of the upconverted photoluminescence emissions by Cu_2_O, and the subsequent transition of the electrons in the valence band to the conduction band, which photo-catalyze the chemical transformations of ammonia borane (AB) for H_2_ production.

**Figure 5:**
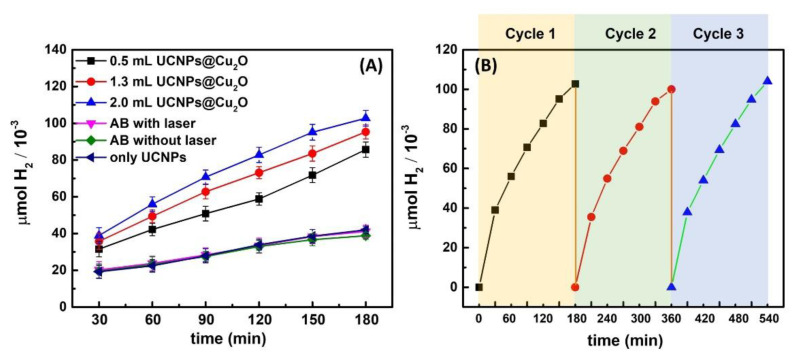
(**A**) H_2_ production profiles from various photocatalytic conditions. Each reaction was kept at 20 °C and irradiated with a 980 nm NIR laser with the power intensity set to 1 W. (The concentrations are 0.476 mg/mL, 1.119 mg/mL, and 1.667 mg/mL for 0.5 mL, 1.3 mL and 2.0 mL of UCNPs@Cu_2_O used, respectively). (**B**) Amount of H_2_ produced over time on repeat cycles of the UCNP@Cu_2_O to show reusability.

The photocatalytic activities of the hybrid NaGdF_4_:Yb^3+^/Er^3+^@NaGdF_4_@Cu_2_O (CSUCNPs@Cu_2_O) nanostructures were investigated by H_2_ evolution from the hydrolysis of AB (NH_3_BH_3_) under 980 nm NIR laser irradiation. The experimental setup was shown in Figure S4. As shown in Figure 5A, the photocatalytic H_2_ production was quantified under various conditions by gas chromatography (GC) equipped with a TCD detector, including pure AB molecules irradiated by 980 nm NIR laser, pure AB molecules without irradiation of 980 nm NIR laser, bare UCNPs with excitation of 980 nm NIR laser, and AB molecules in the presence of three samples of NaGdF_4_:Yb^3+^/Er^3+^@NaGdF_4_@Cu_2_O (UCNPs@Cu_2_O), with increasing volumes (0.5 mL, 1.3 mL and 2.0 mL) under illumination of 980 nm laser. It was demonstrated that the dehydrogenation of AB molecules barely occurred, with negligible amounts of H_2_ being generated in the absence of photocatalysts (NaGdF_4_:Yb^3+^/Er^3+^@NaGdF_4_@Cu_2_O), with and without 980 nm NIR laser irradiation, for a period of 3 h. In addition, slight reaction occurred in the presence of UCNPs without Cu_2_O shells (NaGdF_4_:Yb^3+^/Er^3+^@NaGdF_4_), further confirming the stability of AB molecules in the photocatalytic experiments. The hydrolysis of AB was efficiently catalyzed by photocatalysts (NaGdF_4_:Yb^3+^/Er^3+^@NaGdF_4_@Cu_2_O), and considerable amounts of H_2_ were evolved and detected during the first hour of 980 nm NIR laser irradiation. The production of H_2_ significantly increased over a period of 180 min under the excitation. The enhanced photocatalytic performance can be primarily ascribed to the energy transfer from UCNPs to the Cu_2_O shell and the photoinduced charge carriers promoting the dehydrogenation of AB molecules under NIR excitation. With increasing amounts of NaGdF_4_:Yb^3+^/Er^3+^@NaGdF_4_@Cu_2_O (0.5 mL, 1.3 mL, and 2.0 mL), the photocatalytic H_2_ production was boosted, indicated by the rise in the amounts of H_2_ detected at each 30 min time intervals (Figure 5A). More importantly, core-shell NaGdF_4_:Yb^3+^/Er^3+^@NaGdF_4_@Cu_2_O nanoparticles exhibited exceptional NIR light-driven catalytic durability over multiple cycles of photocatalytic H_2_ evolution (Figure 5B). It can clearly be seen that the photocatalysts suffered little to no loss of activity after three cycles, lasting 540 min, allowing for potential use in an onboard hydrogen application due to the well-preserved photocatalytic activities.

**Figure 6:**
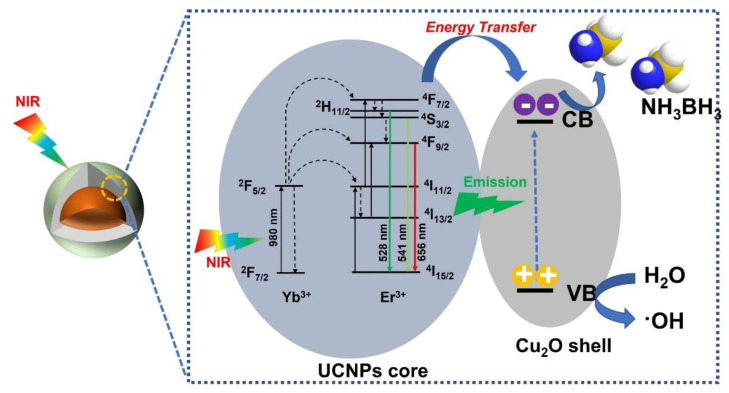
The mechanistic scheme for the transfer of energy from a source, through the particles, to the degradation of ammonia borane.

A schematic of the charge transfer process was proposed to illustrate the NIR-driven mechanism of photocatalytic H_2_ evolution over the synthesized core-shell NaGdF_4_:Yb^3+^/Er^3+^@NaGdF_4_@Cu_2_O hybrid nanostructures in Figure 6. Under the excitation of 980 nm NIR laser, three successive energy transfers (^2^H_11/2_ → ^4^I_15/2_, ^4^S_3/2_ → ^4^I_15/2_ and ^4^F_9/2_ → ^4^I_15/2_) from the sensitizer Yb^3+^ ions to activator Er^3+^ ions occur, resulting in the emissions in ultraviolet (UV) and visible regions. These upconverted emissions are then reabsorbed by Cu_2_O via an energy transfer process to generate photoexcited charge carriers. The band gap value of UCNPs@Cu_2_O was determined to be ~2.25 eV according to the Tauc Plot (Figure S5). The photo-generated electrons are excited from the valence band (VB) to the conduction band (CB) of Cu_2_O, while positively charged holes are left in the VB to react with H_2_O molecules to form hydroxyl radicals. The intermediate of hydroxyl radicals is crucial in the investigation of the mechanism for the photocatalysis, and various methods were reported for the detection [92,93,94,95]. The formation of hydroxyl radicals was confirmed by the addition of 2-propanol acting as the hydroxyl radical scavenger during the photcatalytic H_2_ evolution under 980 nm NIR laser irradiation. The photocatalytic activities significantly decreased for AB dehydrogenation in the presence of 2-propanol, due to the decrease of the concentration of hydroxyl radicals (Figure S6). Due to the electronegativity difference between B and N atoms, the polar and weak B-N chemical bond formed by sharing lone pair electrons between NH_3_ and BH_3_ moieties is susceptible to be attacked by both H_2_O molecules and photogenerated electrons. In addition, during the transformation of as-synthesized hydrophobic UCNPs to hydrophilic ones, the surface of particles was capped by BF_4_^−^ species. These negatively charged species were carried over to the surface of core-shell UCNPs@Cu_2_O hybrid nanostructures, which facilitates the absorption of AB by the electrostatic interaction between the electron-deficient BH_3_ moiety of the AB molecules and BF_4_^−^ species, forming an activated complex species. We assume that the hydroxyl radicals and photoinduced electrons are both reactive species to enhance the catalytic H_2_ evolution for AB dehydrogenation, by dissociating the weak B-N bond in aqueous medium.

## 4. Conclusions

In summary, the novel ß-phase NaGdF_4_:Yb^3+^/Er^3+^@NaGdF_4_@Cu_2_O core-shell hybrid nanoparticles were synthesized for the first time and successfully characterized with a concentration of 10 mg/mL. The intimate contact of the semiconducting shell to the core nanostructures led to an efficient energy transfer from the UCNPs to the Cu_2_O under the excitation of a 980 nm laser, which subsequently generated photoinduced charge carriers actively participating in photocatalysis. The remarkable NIR-driven photocatalytic performance was then evaluated by H_2_ evolution from the dehydrogenation of AB (NH_3_BH_3_) molecules under 980 nm laser irradiation. Upon reaction with AB molecules, the amount of H_2_ produced drastically increased in the presence of the hybrid photocatalysts based on UCNPs and the semiconductor Cu_2_O, compared to pure AB, with and without irradiation, as well as in conjunction with UCNPs without a semiconductor shell. In addition, the NIR-driven photocatalytic activities were highly stable over multiple uses in the recycling experiments. We speculate that this study can serve as the guideline for the rational design and development of NIR-responsive photocatalysts and provide a new direction for improving efficient near-infrared-activated photocatalytic H_2_ production toward sustainable energy utilization in the near future.

## Data Availability

The data presented in this study are available on request from the corresponding author.

## Supplementary Materials

The following are available online at https://www.mdpi.com/article/10.3390/nano11123237/s1, Figure S1: Additional high-resolution TEM image of UCNPs@Cu_2_O core-shell hybrid hetero-nanostructures. Figure S2: XRD patterns of NaGdF_4_: Yb^3+^/Er^3+^@NaGdF_4_@Cu_2_O (UCNPs@Cu_2_O). Figure S3: Energy-dispersive X-ray (EDX) spectra of core-shell NaGdF_4_: Yb^3+^/Er^3+^@NaGdF_4_@Cu_2_O (UCNPs@Cu_2_O) nanoparticles. Figure S4: The picture of the experimental setup for the photocatalytic H_2_ evolution. Figure S5: Tauc plot of UCNPs@Cu_2_O and UV-absorbance of UCNPs@Cu_2_O core-shell hybrid hetero-nanostructures. Figure S6: The photocatalytic H_2_ evolution from AB dehydrogenation over the time catalyzed by UCNPs@Cu_2_O in the absence (black) and presence of 2-propanol (red) acting as the hydroxyl radical scavenger under 980 nm NIR laser irradiation.
