# Supplementary material for: Efficient Near-Infrared-Activated Photocatalytic Hydrogen Evolution from Ammonia Borane with Core-Shell Upconversion-Semiconductor Hybrid Nanostructures
Source: Nanomaterials (Basel). 2021 Nov 29;11(12):3237. doi: 10.3390/nano11123237 (PMC8707141; doi:10.3390/nano11123237)
Supplement: Supplementary file 1 [file nanomaterials-11-03237-s001.zip › nanomaterials-1490048-supplementary.pdf]

Supplementary Materials

Efficient Near-Infrared-Activated Photocatalytic Hydrogen Evolution from Ammonia Borane with Core-Shell Upconversion-Semiconductor Hybrid Nanostructures

Andrew J. Evangelista, Mariia Ivanchenko and Hao Jing *

Department of Chemistry and Biochemistry, George Mason University, Fairfax, VA 22030, USA; aevange3@gmu.edu (A.J.E.); mivanch@gmu.edu (M.I.)

* Correspondence: hjing2@gmu.edu

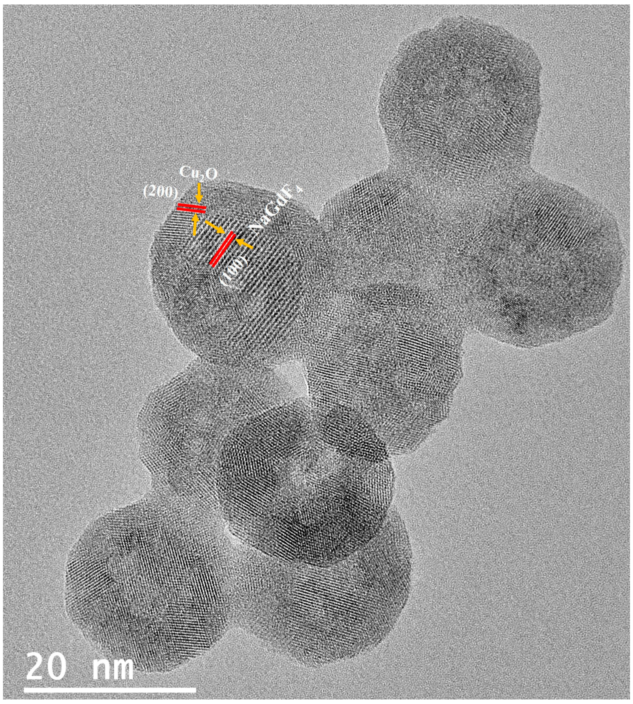

Figure S1. Additional high-resolution TEM image of UCNPs@Cu_2O core-shell hybrid hetero-nanostructures.

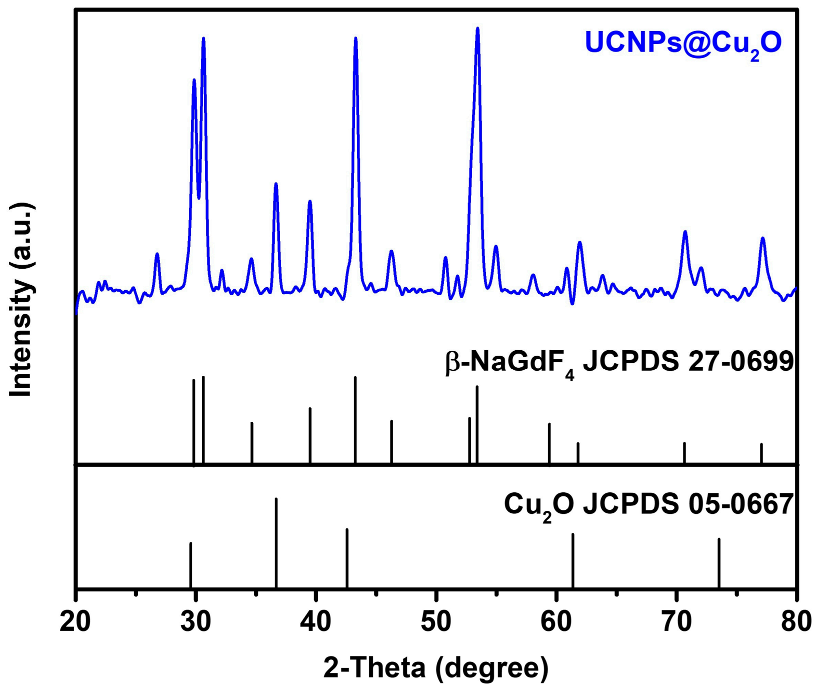

Figure S2. XRD patterns of $NaGdF_4$:Yb^{3+}/Er^{3+}@$NaGdF_4$@Cu_2O (UCNPs@Cu_2O). Standard XRD patterns of pure $NaGdF_4$ (JCPDS 27-0699) and Cu_2O (JCPDS 05-0667) are also shown.

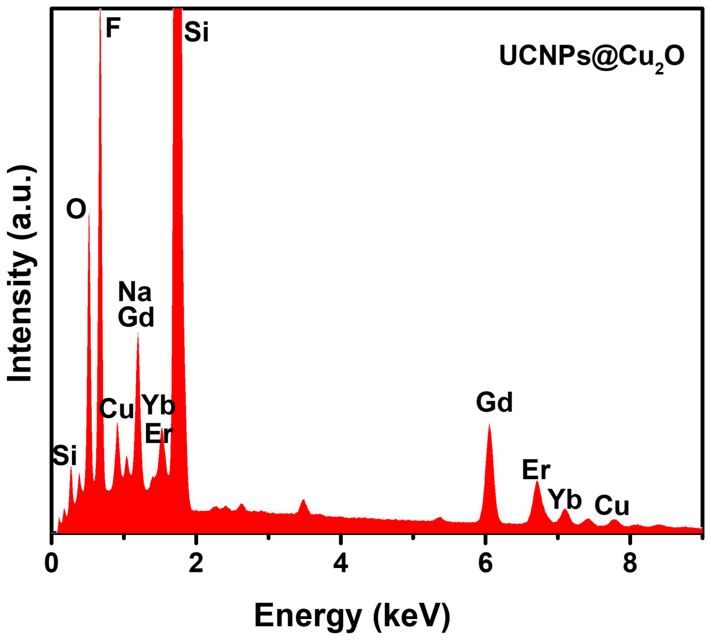

Figure S3. Energy-dispersive X-ray (EDX) spectra of core-shell $NaGdF_4$:Yb^{3+}/Er^{3+}@$NaGdF_4$@Cu_2O (UCNPs@Cu_2O) nanoparticles.

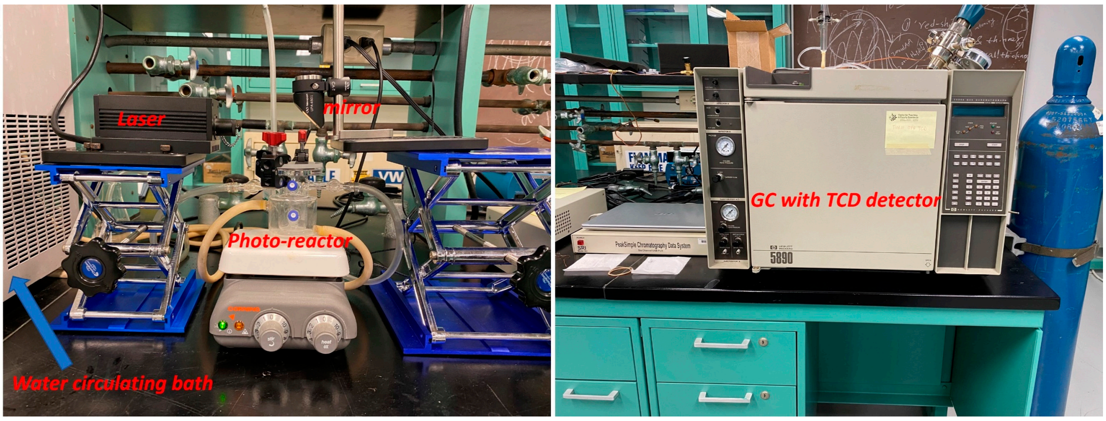

Figure S4. The picture of the experimental setup for the photocatalytic H_2 evolution.

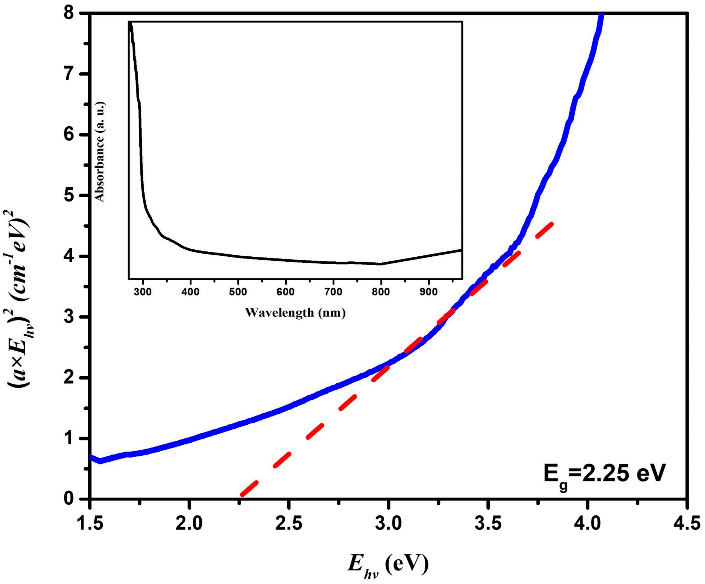

Figure S5. Tauc plot $\alpha h\nu = A(h\nu - E_g)^{1/2}$ of UCNPs@Cu_2O, where α is the absorption coefficient, h is the Plank constant, ν is the photon frequency, and A is a constant. Direct band gap values are determined by extrapolating $h\nu$ to $\alpha = 0$. (Inset) UV-absorbance of UCNPs@Cu_2O core-shell hybrid hetero-nanostructures.

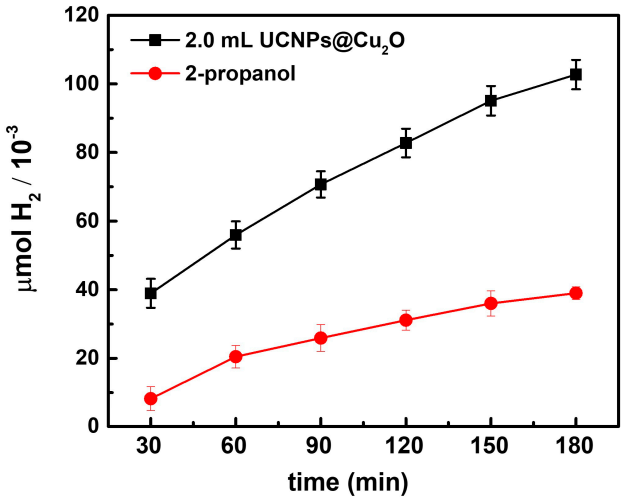

Figure S6. The photocatalytic H_2 evolution from AB dehydrogenation over the time catalyzed by UCNPs@Cu_2O in the absence (black) and presence of 2-propanol (red) acting as the hydroxyl radical scavenger under 980 nm NIR laser irradiation.
